# The Amalgam Question

**Published:** 1861-07

**Authors:** 


DENTAL REGISTER OF THE WEST.

VOL. XV.] JULY, 1861. [No. 7.

### Original Essays and Communications.

### THE AMALGAM QUESTION.

WE promised, when we felt like it, to say something on the "Amalgam" discussions in the Pennsylvania Association of Dental Surgeons. We are reluctant to say anything; for our brethren of the said association appear to delight in the luxuries of incredulity in regard to some of the plainest teachings of science, and it is a pity to mar their enjoyments. Incredulity is a nice thing sometimes, and sometimes belief is a bore, and faith a fancy.

Although the fact of metallic poisoning is as well established as any other, yet all classes incline to disbelieve it to the extent of their own personal interest in its truth. The painter believes that the oxyds and salts of lead are poisonous, and that many of his craft are injured by them; but he does not believe that he is in any danger. He regards himself as cautious and cleanly, and, therefore, safe. He may believe that his comrades are poisoned, but he is not. He is only a little nervous, and is rather costive, or has eaten something indigestible, and has colic. The margins of his gums are bluish; but that is because his blood circulates badly. He has seen many such cases; and to prove that lead has "nothing to do with the trouble existing," he keeps right on at his business. The printer believes that both lead and antimony are poisonous; but he handles the types with a thorough conviction that they will not poison him. When he becomes nervous and pale, he thinks it *possible* that *confinement* disagrees with him; and, though his pallor is not like that of other pale men, he imagines that printer's ink may cause the difference. Gilders, miners, and even chemists, practically incredulous while theoretically believing, often reason in the same way, and it is not, therefore, marvelous that our brethren of the Pennsylvania Association are practically incredulous in regard to the poisonous effects of mercury and silver. This is one way by which they manifest their humanity. "Ye shall not surely die" was a popular doctrine, as far back as the days of Mother Eve's horticultural experience.

On the evening of April 9th, 1861, as we learn by the May number of the *Cosmos*, the President of the association announced "Amalgam" as the subject for discussion. Dr. Pierce opens cautiously, manifesting a desire to speak favorably of the article under consideration, but seems fearful of misrepresentation by those who "are constantly attributing results to it that are due, not to the amalgam, but to its improper use," and "those who, unable to perform a successful operation with gold, are eager to seize upon any remark in favor of a plastic material as an apology for their own empirical conduct." This last is certainly worthy of consideration; for all quackdom shouts at the very thoughts of the May number of the *Cosmos*. It matters not to the quack that the said number contains much valuable matter. It contains an endorsement of his pet paste by the Pennsylvania Association of Dental Surgeons. That is glory enough for him; and he drinks in consolation accordingly.

The report, as a whole, is an interesting specimen of special pleading in behalf of the material under consideration—

"Only this, and nothing more,"—

yet it is amusing to see how little these brethren can find to say in its favor, and how little they really think of it, notwithstanding their eulogies.

A frontier preacher conducting the funeral services of a notorious outlaw, politeness and truth being both desirable, was at a loss what to say. "My friends," said he, "our deceased brother, like all things earthly, was perhaps not just all that he should have been. It is true he had horses, and he ran 'em. He had cocks, and he fit 'em. He had cards, and he played 'em. But after all, he was a very good man at a fire; and no man in these diggins ever cheated him in a hoss-trade." About such, when sifted, is the praise bestowed by our brethren on amalgam.

Dr. Buckingham uses it "*sparingly.*" Dr. McQuillen has a hopeless prejudice against its use. Early prejudices have led Dr. Fitch "to look with considerable suspicion upon amalgam as being, in any sense, a proper material for filling all classes of teeth." Dr. Flagg once had a severe attack of "the prevailing prejudice" against it, but has quite recovered. In six years he has seen amalgam plugs "in many hundred mouths," which proves that vice seen too oft, if not pitied, is at least embraced. Dr. McGrath never was "imbued with any prejudices against" it. He escaped the disease entirely; and that, too, it seems, without vaccination.

It appears that none of these brethren use amalgam indiscriminately. Dr. Peirce, under no circumstances, would use it in the front teeth, "believing that the preservation of their beauty is as essential as subserving the purpose of nutrition;" and he tells us that "in the largest proportion of teeth that are filled with it, their good appearance is completely sacrificed." Dr. Buckingham uses it only "in certain cases;" but the report fails to tell us why he repudiates it in *uncertain* ones. Dr. McQuillen would use it only in "teeth which it would be impossible to fill with gold foil, and yet too valuable to sacrifice." He would not use it in all, because he can not "entirely free himself from such prejudice, so as to use it indiscriminately," and because "it not only required more skill, but also afforded one more satisfaction to introduce a good gold filling, and such practice was by far better calculated to enable one to establish a high reputation as an operator." These reasons are, no doubt, good and valid, in the present case, but if any operator is already freed from "such prejudice," and his reputation is as high as is desirable, he may, possibly, find as much satisfaction in using amalgam indiscriminately, as in introducing good gold fillings, and if it is important that he should exercise skill, for the mere sake of skill, he can spend the time gained by such indiscriminate use in stuffing butter into a gimlet hole "with a hat awl." When prejudice restricts the use of anything so valuable as amalgam appears to be in the Pennsylvania Association, it should be laid aside.

Dr. Fitch seldom uses amalgam, "and then never in any but molar teeth very much decayed." Now why? Dr. Fitch. Do tell us. Dr. F. claims that it can be readily adapted to the walls of the cavity so as "to *exclude* all foreign destructive agencies"—that the objectionable discoloration is probably due to "the impurity of the materials, or to the presence of their oxyds, or the manner of their combination." He infers all this from the fact that he finds "no free or combined acids" in the mouth capable of acting "to any extent upon pure mercury or silver." Indeed! But does Dr. F. not know that oxygen and sulphur, both found in the mouth, act directly on both these metals, without the intervention of an acid? Moreover, he tells us that the mercury of commerce is "quite impure, and the silver usually very much oxydized at the time of using." Well, really! The secretions of the mouth can not act on the silver, but something (in the atmosphere, perhaps,) oxydizes it so readily that oxydized silver is generally used in preparing amalgam! And, again, he tells us that "coloration may be due to galvanic action, induced by the saline secretions of the mouth, in the oxydation of one of the baser metals." Tell us, Dr. F., which two of the metals are *baser*, and then we will know which *one* is base. And tell us, too, how to arrest the galvanic action, when there are "saline secretions" in the mouth, before it has time to produce coloration. And tell us what kind of people, and in what kind of weather they do not have "saline secretions in the mouth."

Dr. McGrath uses amalgam whenever he thinks it "necessary." Good! for him. He can pass; for that is the way we do. But we have never used it yet.

Dr. Flagg "does not use amalgam *from choice*, in any teeth except molars." And we protest that it is very wicked for anybody to *compel* him to use it in any others. He used to have two objections to its use; but by the help of Dr. Garretson, and *some salt*, "he had been able, in a great measure, to do away with" one of them. The remaining one is "the failure to preserve the teeth in plugging certain cavities," which is rather a serious objection, and one beyond the reach of salt, we fear. Dr. F. finds that amalgam fillings on the proximal surfaces of teeth fail "in a comparatively short time," while upon the articulating faces of molar teeth, they do "better service than anything except the very best gold plugs, and equaling even these, as far as six years' proving could testify." And in comparing amalgam with gold for filling such cavities, especially when large, "he thought the argument was decidedly in favor of amalgam; for while it saved teeth as permanently as the very best gold plugs, it was introduced with much less of disagreeable manipulation, less irritation, less consumption of time, less expense, and was so nearly the color of the tooth as to be scarcely noticeable." Now, if these claims are all well founded, gold should be abandoned; for, if amalgam is as good as gold in large cavities, it is better in small ones, and if decidedly preferable on articulating surfaces, it is certainly as good in proximal cavities; for it is as much exposed to chemical action in the one place as in the other. And if it is so nearly the color of the tooth as to be scarcely noticeable, it is the very thing for front teeth, and should, by no means, be restricted to molars. And if it is so reliable that in six years it is affected just none at all by chemical action, in twelve years it will be affected only twice as much, which will not be serious. We protest that the friends of amalgam treat it shabbily. Come, now! Give it a fair chance. If it saves the frail teeth with the large cavities—if it saves the unfavorable cases as well as gold does the favorable ones, what wouldn't it do in firm teeth with small cavities? It would heal up the holes and "hair them over" in less than a week.

But the discoloration—that's the bugbear—the hobgoblin that haunts the imaginations of our friends of the Pennsylvania Association. We are all superstitious—are the worst frightened at that which is the most mysterious. And our friends (most of them, at least,) appear to be in a quandary as to the causes of the discoloration, and are, therefore, more concerned about the looks of a tooth than the health of a patient. Dr. Flagg comes to the rescue, however, and the ghost is laid. He removes and prevents the discoloration, by triturating the amalgam with common salt, and afterward washing it. "Salt is good." Blessed be the man that invented it! A cenotaph to his memory! Who heads the subscription? But, poor man, he didn't know it all. He knew how to "save his bacon;" but our friend can keep a nastier thing than swine's flesh from spoiling. May his countenance be perpetuated in marble. Who'll *bust* him?

But we ought to be serious over this salt and water process, and would be, if it amounted to anything more than a mere removal of the oxyds necessarily formed during combination, which can be accomplished as effectually, and almost as readily, with water or alcohol.

These brethren of the Pennsylvania Association use, substantially, the preparation called "Townsend's Amalgam;" though just why it is called *Townsend's* is hard to divine, since Dr. T. set up no claim to originality in regard to its preparation, but obtained the formula, we are sorry to say, in the West. But there is a funnier thing than this. Why did not Dr. Townsend obtain as satisfactory results with this amalgam as these brethren report, when he prepared and introduced it as carefully and perfectly as any can claim to do now? Why did he obtain such results that he found it necessary to make a public recantation of almost everything he ever claimed for it? Was he entirely mistaken about it when he said that in cases where he most relied upon it, and expected to have the best results, it entirely failed? And why should not these brethren, who so blindly followed Dr. T. when he went astray, follow him back, when he so promptly and manfully returned to the paths of scientific truth? We protest that if the sheep do jump into the sea, because their blinded bell-wether falls over the cliffs, if he bravely swims out and returns to the pasture, they ought to follow him.

Amalgam cement used to be made of mercury and silver. Various other metals were recommended as ingredients. The old silver amalgam long since had become very unpopular. It was regarded as a *black spot* on our professional escutcheon. And what is this that is to be dubbed respectable? Is either of the base metals of the old formula discarded? Not at all. They are simply rendered respectable and perfect by the addition of *another base metal!* An ox-driver, who fancied his team was not just the ton, hitched a mule in front of them, and concluded all was right; but he found that his cart was still a *slow coach.* So these brethren, not satisfied with silver and mercury, add *tin;* but they find the hated black spot still there, in spite of all their tin-kering.

Dr. Flagg tells us—or rather the report tells us that the Doctor "remarked that the discoloration of amalgam was superficial, and that it was oftentimes possible to restore the beauty of a tooth by simply removing the amalgam plug and excavating the darkened portion of dentine." That might answer, sometimes, and is worthy of attention. The idea has quite a range of application. If the entire tooth becomes discolored, pull it out,—if the entire head, cut it off; and if the patient's complexion becomes darkened by the oxyd of silver, the discoloration, being "superficial," can be removed by "simply" skinning him.

This paper is already too long. We will, therefore, close; and, if in the mood, we will, at another time, say something in regard to the causes of discoloration, so annoying to our friends, and perhaps notice some of their answers to objections made against the use of amalgam cements. We look upon this report as calculated to becloud and lead astray the minds of the more youthful in our profession, and this is our only apology for bestowing on it so much more attention than it really merits. W.

